# Factors contributing to variability in neurocognitive performance before glioma neurosurgery

**DOI:** 10.1093/nop/npae106

**Published:** 2024-10-20

**Authors:** Izabelle Lövgren, Natalie Laura Voets, Claire Isaac, Susan Isabel Honeyman, Juan Felipe Mier, Richard Stacey, Vasileios Apostolopoulos, Puneet Plaha

**Affiliations:** Wellcome Centre for Integrative Neuroimaging, FMRIB, Nuffield Department of Clinical Neurosciences, University of Oxford, Oxford, UK; Wellcome Centre for Integrative Neuroimaging, FMRIB, Nuffield Department of Clinical Neurosciences, University of Oxford, Oxford, UK; Department of Neurosurgery, Oxford University Hospitals NHS Foundation Trust, Oxford, UK; Russell Cairns Unit, John Radcliffe Hospital, Oxford University Hospitals NHS Foundation Trust, Oxford, UK; Department of Neurosurgery, Oxford University Hospitals NHS Foundation Trust, Oxford, UK; Department of Neurosurgery, Oxford University Hospitals NHS Foundation Trust, Oxford, UK; Department of Neurosurgery, Oxford University Hospitals NHS Foundation Trust, Oxford, UK; Department of Neurosurgery, Oxford University Hospitals NHS Foundation Trust, Oxford, UK; Department of Neurosurgery, Oxford University Hospitals NHS Foundation Trust, Oxford, UK; Nuffield Department of Surgical Sciences, University of Oxford, Oxford, UK

**Keywords:** cognition, glioma, health-related quality of life, neurosurgery

## Abstract

**Background:**

Cognitive impairment following anti-tumor treatment is a common concern for brain tumor patients. However, growing evidence indicates that significant impairments can be present even before treatment. The purpose of this study was to identify factors that explain variability in pretreatment test performance, beyond that of tumor burden.

**Methods:**

Using multi-step linear regression, we retrospectively probed the contribution of clinical-, tumor-, patient-, and self-reported factors to variance in performance among 96 treatment-naïve brain tumor patients across 13 objective neurocognitive tests. Agreement between subjective and objective reports of cognitive impairment was also examined.

**Results:**

Clinically significant preoperative impairments were observed in both objective and subjective domains. Estimated premorbid intelligence quotient (IQ), tumor volume, diagnosis of an astrocytoma, self-reported depression, and perceived cognitive functioning scores were the most common predictors of objective neurocognitive performance prior to treatment, explaining 12.3%–58.3% of the variance. No association was identified between objective and subjective reports of cognitive impairment.

**Conclusions:**

Glioma patients frequently exhibit objective and subjective impairments prior to treatment. Both tumor- and self-reported factors were identified as predictors of performance, after correcting for estimated premorbid IQ. Nevertheless, more than 41.7% of the variance in cognitive performance remained unexplained, indicating a substantial role for additional, as yet unaccounted for, clinical factors. Notable disparity between objective and subjective cognitive impairment status re-emphasizes the importance of assessing both domains to ascertain a patient’s overall functioning in the context of treatment outcomes.

**Key Points**

- This study highlights the additional contribution of clinical-, patient-, and self-reported factors in explaining the variance in pretreatment cognitive functioning of brain tumor patients, beyond that of tumor burden. It also emphasizes the discrepancy between objective and subjective impairment status.
- In addition to identifying factors for future investigations, these findings reinforce the need for widespread, systematic cognitive assessment of glioma patients. Nevertheless, further refinement of standard cognitive tests may be needed to improve their sensitivity to subjective quality-of-life complaints, which are central to informing optimal treatment strategies.

**Importance of the Study**

Historically, extensive focus has been placed on the effects of treatments such as surgery and radiochemotherapy on cognitive performance in brain tumor patients. However, beyond the impact of the tumor itself, little is known about what drives variability in cognitive functioning prior to starting treatment. Our findings emphasize that significant objective and subjective impairments are common among glioma patients even before treatment begins. In addition to tumor burden, we identify key factors that further affect cognitive performance (eg, dexamethasone treatment for brain swelling, mood ratings). Furthermore, we observed a clear disparity between objective and subjective impairment status, stressing that the absence of subjective complaints should not discourage referrals for neuropsychological evaluation. As treatments increasingly prolong survival, a comprehensive understanding of the factors that affect patient performance is central to maximizing long-term quality of life.

Brain tumors contribute significantly to morbidity, mortality, and healthcare-associated costs.^1–3^ Gliomas, originating from glial cells, account for most primary brain malignancies in adults.^1,4^ While low-grade gliomas confer a more favorable prognosis, malignant transformation into a high-grade tumor may occur within an unknown time frame. Consequently, clinical management of glioma is focused on stemming tumor growth and palliating symptoms.^5^

Impairments in cognitive function and health-related quality of life (HRQoL) are common in glioma patients.^6–9^ While these deficits have predominantly been evaluated as treatment-related side effects, many patients exhibit significant impairments in cognitive functioning and/or HRQoL prior to anti-tumor treatment.^10,11^ One systematic review^12^ reported a median incidence of impairment in any cognitive domain of 62.6% in treatment-naive glioma patients, with executive function, psychomotor speed, and memory being most commonly affected.^12^ Given the infiltrative nature of gliomas, pretreatment deficits are primarily attributed to tumor-related factors (mass effect, location, histopathological profile). Nevertheless, there is clear heterogeneity regarding which patients experience clinically significant impairment prior to treatment, as some patients appear to exhibit greater resilience to the effects of the tumor.^1^ To contextualize postsurgical outcomes and identify avenues for intervention, a better understanding of the factors driving this pretreatment variance is needed.

Few studies^6,8,13^ have probed the contribution of patient-related factors to pretreatment performance (eg, premorbid ability, medical history, and other factors potentially captured via self-reported scales). The utility of self-reported scales is complicated by reports of weak correlations between self-reported cognitive function and objective performance in glioma patients.^5,7^ Gehring et al.^7^ postulated that this disparity may be driven by under-reporting of cognitive problems in individuals with substantial objective impairments (due to a lack of awareness), coupled with over-reporting by patients also experiencing psychological symptoms. Hence, associations between self-reported and objective complaints warrant further investigation.

Here, in a cohort of treatment-naive glioma patients, we aimed to (1) report incidences of pretreatment impairment across thirteen objective neurocognitive tests plus self-reported scales (perceived function, mental health, fatigue); (2) evaluate whether tumor-, clinical-, patient-, and self-reported factors contribute to variance in pretreatment objective cognitive performance; and (3) probe the agreement between objective and subjective cognitive *impairment*. In addition to this, we report the incidence of significant postoperative complaints (*n* = 45) and significant changes in objective performance (*n* = 44) in a smaller subset of patients with radiologically stable disease (Supplementary Section 1).

## Methods

### Design

We performed a retrospective analysis of treatment-naive glioma patients who underwent preoperative outpatient neuropsychological evaluation at the Oxford University Hospitals (OUH) NHS Foundation Trust up to March 5, 2024. Preoperative assessments are part of routine work-up for all our low-grade glioma patients, and typically those with high-grade gliomas affecting the presumed language-dominant hemisphere. The study was approved by the Oxford B Research Ethics Committee (Reference:13/SC/0553).

### Participants

We identified 122 individuals with available neuropsychological assessments. Twenty-six were excluded due to previous neurosurgery (other than biopsy, *n* = 8), radio/chemotherapy (*n* = 6); nonfluent English speaker (*n* = 15); unconfirmed language lateralization in left-handed patients (*n* = 2); callosal agenesis (*n* = 1), invalid neuropsychological scores (*n* = 1), and confounding learning disability (*n* = 1). Nonfluent speakers were excluded due to the heavy reliance of neurocognitive assessments on verbal skills and cultural familiarity with test items.^14^ Indeed, sub-analysis highlighted significantly worse performance of nonfluent English speakers compared to native speakers across tests. Our final cohort comprised 92 patients with histopathologically confirmed glioma and 4 patients with a radiologically appearing glioma, whose tissue analysis was not diagnostic (brain tumor, “not otherwise specified” [NOS]). In right-handed patients (*n* = 86), left hemisphere language dominance was assumed or inferred from presenting language symptoms. In 7/10 left-handed patients, hemisphere of language dominance was confirmed using functional magnetic resonance imaging (MRI) and/or awake intraoperative mapping. As language dominance was not confirmed in the 3 remaining patients (each with a right-hemisphere tumor), their scores were excluded from analyses evaluating the effect of language-dominant hemisphere involvement.

### Neuropsychological Evaluations

Neurocognitive tests and subjective self-report scales (Table 1) were administered by a Consultant Clinical Neuropsychologist, and the most systematically used assessments are reported.

**Table 1.** Neuropsychological Tests and Subtests Evaluated

Evaluation	Domain	Battery^*^	Test
Objective	Language	- WAIS-IV BMIPB	*Boston Naming Test* *Similarities* *Semantic Verbal Fluency*
	Verbal memory Visual memory	BMIPB BMIPB	*List Recall* *Figure Immediate Recall* *Figure Delayed Recall*
	Verbal Learning	BMIPB	*List Learning*
	Executive Function	D-KEFS WAIS-IV FAS Test	*Colour Word Interference: Inhibition (Stroop interference).* *Colour Word Interference: Inhibition/Switching (Stroop Switching).* *Digit Span* *Phonemic Verbal Fluency*
	Nonverbal reasoning	WAIS-IV	*Matrix Reasoning*
	Information Processing	BMIPB	*Speed of Information Processing*
	Premorbid ability (IQ)	- -	*National Adult Reading Test* *Test of Premorbid Function*
Self-reported	Health-related Quality of Life	EORTC QLQ-C30	*Cognitive Functioning* *Emotional Functioning* *Role Functioning* *Physical Functioning* *Social Functioning* *Fatigue*
	Mental Health	HADS	*Anxiety Score* *Depression Score*

Abbreviations: WAIS-IV = Wechsler Adult Intelligence Scale Fourth Edition^15^; FAS test^16^; BMIPB = The Brain Injury Rehabilitation Trust Memory and Information Processing Battery^17^; CVLT = California Verbal Learning Test^18^; D-KEFS = Delis-Kaplan Executive Function System^19^; IQ = intelligence quotient; EORTC QLQ-C30 = EORTC Core Quality of Life questionnaire^20^; HADS = Hospital Anxiety and Depression Scale.^21^.

^*^If the test is a subset of a wider battery.

#### Objective Cognitive Performance.—

Neuropsychological subtests evaluated executive functioning, verbal and visual memory, speed of information processing, verbal reasoning, and nonverbal perceptual reasoning. For each test, *z*-scores were calculated using age-specific, normative data. As per routine clinical practice, education level was also accounted for in the Phonemic Verbal Fluency test and Boston Naming Test (BNT). Patients were considered “impaired” if their *z*-score was ≤ −1.5, consistent with previous literature.^5,13,22^ Base-rates of impairment are reported at this cutoff, as well as an alternate, more conservative cutoff of *z* ≤ −2. In addition to this, the incidence of deficits in 2 or more objective cognitive tests is also reported, to provide a more comprehensive overview of the extent of disruption experienced, beyond that of a single test.

#### Self-reported Scales.—

From 2016, neuropsychological assessments were supplemented with quality-of-life questionnaires. Accordingly, 53 patients also completed European Organization for Research and Treatment of Cancer Quality of Life Questionnaire-C30 (EORTC QLQ-C30).^20^ From these, 5 “Functioning” scale scores were evaluated, alongside Fatigue from the symptom scale. In addition, 75 patients completed the Hospital Anxiety and Depression Scale (HADS).^21^ Thresholds for clinically significant impairments were based on previous reports (see Supplementary Section 2).^21,23^

#### Tumor Volume Calculation.—

Tumor volume was calculated either manually, or through a combination of (semi-) automatic segmentation supplemented with manual review. Ring-enhancing high-grade tumors were segmented on the post-Gadolinium T1-weighted MRI, including all contrast-enhancing tissue but excluding peri-tumoral edema. Nonenhancing tumors were segmented on the fluid attenuated inversion recovery (FLAIR) sequence, including all hyper-intense tumor signal, but excluding peri-tumoral edema. Tumors with more complex appearances (including enhancing and nonenhancing tumor or heterogeneous nonenhancing tumor) were delineated on the most representative scan sequence (eg, T1 noncontrast) with reference to the other sequences (eg, T2/FLAIR/T1 contrast). Heterogeneous tumors were defined manually or using a combination of manual and semi-automated segmentation modes in the software ITK-SNAP.^24^ Ring-enhancing tumors and “low-grade” gliomas were typically segmented using the automated software Raidionics,^25^ with manual correction where needed.

### Factors Affecting Pretreatment Performance

Potential predictors (Table 2) were informed by a previous systematic review^6^ and also included additional, selected variables. Medical history burden was represented as the number of comorbidities reported in the surgical notes and includes both psychiatric and medical comorbidities. As seizure history and use of anti-seizure medication were represented as independent variables, seizures/epilepsy was not included in the medical history burden count. To identify factors that explain variance in pretreatment neurocognitive test scores, we adapted the analysis by Röttgering et al.^26^ In brief, multi-step linear regression modeling was performed by combining all variables that were independently associated with the test score in univariable models (*P* < .05) into a multivariable model to evaluate how several factors *together* influence performance. Backward selection (ie, sequential removal of the least useful variable in the model), was then performed until no predictors remained. An illustrative example is provided in Supplementary Section 3.

**Table 2.** Variables Investigated as Potential Predictors of Performance in Objective Cognitive Testing Prior to Anti-tumor Treatment

Factors	Predictors	Format of data
Tumor-related	Tumor volume	cm^3^
	WHO grade	2, 3, 4
	IDH mutation	Present/Absent
	Glioma subtype	Astrocytoma or GBM, Oligodendroglioma
	Primary lobe affected	Frontal, Parietal, Temporal, Occipital
	Involvement of language-dominant hemisphere	Yes/No
	Insular involvement	Yes/No^*^
Clinical	History of any seizures	Yes/No
	History of generalized tonic-clonic seizures	Yes/No
	Levetiracetam use	Yes/No
	Dexamethasone use	Yes/No
	Medical history burden	0, 1, ≥ 2 reported comorbidities^†^
Patient-related	Age	Years
	Sex	Male/Female
	Estimated premorbid IQ	IQ score
Self-reported	Cognitive functioning	Score as %
	Physical functioning	Score as %
	Emotional functioning	Score as %
	Role functioning	Score as %
	Social functioning	Score as %
	Fatigue score	Score as %
	Anxiety score	Raw score
	Depression score	Raw score

Abbreviations: IDH = isocitrate dehydrogenase; IQ = intelligence quotient; WHO = World Health Organization.

^*^Characterized by > 50% involvement.

^†^Reported further in Supplementary Table 1.

The model that yielded the best goodness of fit at any point in the selection process was chosen as the final model, representing the combination of variables that together explained most of the variability in specific cognitive test scores. Final variables were either (1) significant predictors of the test score or (2) not significant predictors but provided meaningful contribution to goodness of fit (ie, their inclusion explained more variance than when they were excluded, after correcting for the number of predictors—see below).

As subjects without complete data for all variables under investigation were excluded from the model, cohort sizes on which each model was based varied. The final cohort size is reported for each model. Adjusted *R*^2^ quantified the goodness of fit of the models, to correct for differences in numbers of predictors. Supplementary Table 2 summarizes the data considered in the final models.

### Agreement Between Objective and Subjective Measures of Cognitive Impairment

To test for significant associations between self-reported and objective cognitive impairment status, we generated contingency tables for subjective impairment (EORTC QLQ-C30 Cognitive Functioning item score < 75%; yes/no) × objective impairment (test *z*-score ≤ −1.5; yes/no) and performed χ2 tests or Fisher’s Exact Tests, as appropriate, for each cognitive test.

Relationships between self-reported cognitive functioning and both self-reported mood (Anxiety, Depression) and Fatigue scores were also evaluated using Pearson’s correlation coefficient.

### Statistics

Descriptive statistics, Pearson’s correlations, and Mann–Whitney *U* tests were performed using SPSS Statistics (V29.0.1.0). Univariate and multivariable linear regression models, χ2 tests and Fisher’s Exact Test were performed in Python (V3.12.2) using Statsmodels^27^ and SciPy,^28^ respectively. The threshold for significance was set at *P* < .05.

### Patient and Public Involvement

Patients and/or the public were not involved in the design, or conduct, or reporting, or dissemination plans of this research.

## Results

The preoperative cohort comprised 54 males and 42 females with a mean age (± SD) of 41.1 ± 12.9 years, of which 62/96 patients (64.6%) had involvement of the language-dominant hemisphere (Supplementary Table 1). The most common medicated comorbidities included in the medical history burden count were depression (*n* = 13), anxiety (*n* = 7), hypertension (*n* = 8), asthma (*n* = 5), diabetes (*n* = 4), and sleep disturbance (*n* = 4). Test scores did not differ between patients who did (*n* = 8) and did not (*n* = 88) have preceding diagnostic biopsy (*n* = 8). Main demographics are presented in Table 3, with additional information available in Supplementary Table 1.

**Table 3.** Demographics of the Preoperative Cohorts

	Preoperative cohort
*N*	96
Age (years), mean ± SD	41.1 ± 12.9
Sex (male:female), *n* (%)	54:42 (56.3%:43.7%)
Tumor type, *n* (%)	
* IDH* ^*mut*^ Astrocytoma	
WHO Grade 2	20 (20.8%)
WHO Grade 3	20 (20.8%)
WHO Grade 4	1 (1.0%)
* IDH* ^*mut*^ Oligodendroglioma	
WHO Grade 2	24 (25.0%)
WHO Grade 3	10 (10.4%)
* IDH* ^*WT*^ Glioblastoma	17 (17.7%)
Not otherwise specified	4 (4.2%)
Tumor location, *n* (%)	
Left Hemisphere	
* Frontal*	23 (24.0%)
* Parietal*	3 (3.1%)
* Temporal*	32 (33.3%)
* Insular (isolated)*	2 (2.1%)
Right Hemisphere	
* Frontal*	23 (24.0%)
* Parietal*	3 (3.1%)
* Temporal*	8 (8.3%)
* Insular (isolated)*	1 (1%)
Both hemispheres	1 (1%)
Insular involvement^a^, *n* (%)	21 (21.9%)
Previous biopsy, Yes: No *n* (%)	8: 88 (8.3%: 91.7%)
Time between preoperative evaluation and surgery, days (median ± IQR)	100.5 ± 212

^a^Characterized by >50% involvement of insular cortex.

### Incidence of Impairment Prior to Treatment

**Figure 1.**
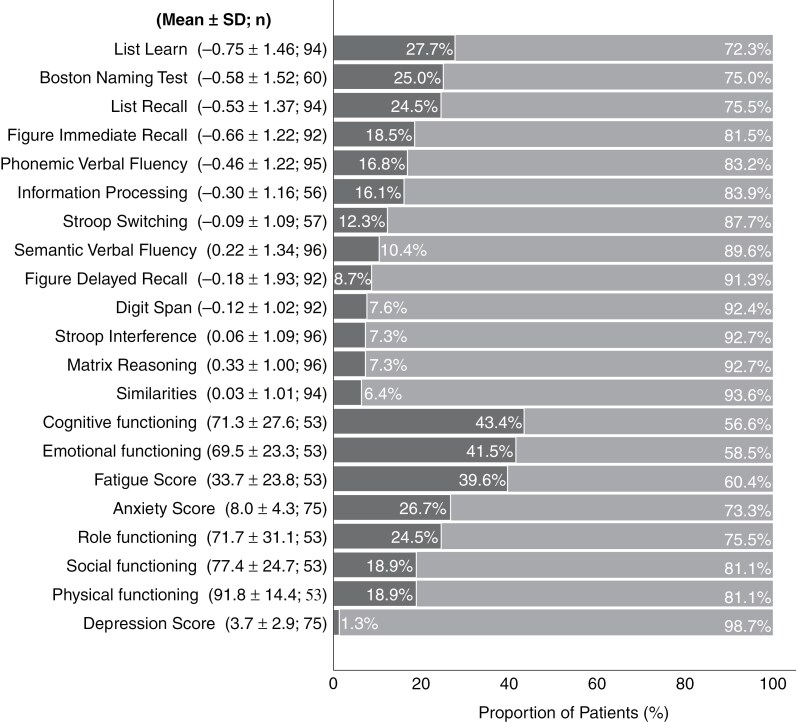
Incidence of impairments in objective cognitive tests and self-reported scales in previously untreated brain tumor patients. The percentage of patients (out of the total available, “n”) that are impaired (darker gray) versus not impaired (lighter gray) are shown, and corresponding mean and standard deviation (SD) of *z*-scores are described. Impairments were observed across all domains. In the objective evaluations, learning, memory, and language function were the most commonly affected domains, followed by executive functioning and information processing. On the self-reported scales, Cognitive and Emotional Functioning, as well as Fatigue Scores showed the greatest incidence of impairment.

Neuropsychological evaluations were performed a median (± IQR) of 100.5 ± 212 days prior to surgery. Preoperative impairments were observed across all neurocognitive tests, with 59/96 (61.5%; *z* ≤ −1.5) and 43/96 (44.8%; *z* ≤ −2) of patients impaired in at least one objective cognitive assessment. Mean ± SD *z*-scores for each test are reported in Figure 1. Deficits in multiple tests were also frequent, with 38/96 (39.6%) having 2 or more *z*-scores ≤ −1.5, and 28/96 (29.2%) scoring ≤ −2 in 2 or more tests. Test-wise rates of impairment at *z* ≤ −1.5 are shown in Figure 1. Deficits were most frequent in verbal learning (List Learning: 26/94; 27.7%), verbal memory (List Recall: 23/94; 24.5%), language (BNT: 15/60, 25%), and visual memory (Figure Immediate Recall: 17/92; 18.5%) at *z* ≤ −1.5. The incidence of impairment organized by the primary brain lobe affected is illustrated in Supplementary Figure 1.

With respect to *z* ≤ −2, rates of impairment were as follows: verbal memory (List Recall: 20/94, 21.3%), verbal learning (List Learning; 19/94, 20.2%); visual memory (Figure Immediate Recall: 12/92, 13.0%; Figure Delayed Recall: 4/92; 4.3%); language (BNT: 9/60, 15.0%; Semantic Verbal Fluency: 4/96, 4.2%; Similarities: 2/94, 2.1%); executive function (Stroop Switching: 5/57, 8.8%; Phonemic Verbal Fluency: 8/95, 8.4%; Stroop Interference: 6/96, 6.3%; Digit Span: 3/92; 3.3%); nonverbal reasoning (Matrix Reasoning: 3/96, 3.1%); and Speed of Information Processing (2/56; 3.6%).

Performance and/or mood complaints were also common across self-reported scales (Figure 1). Impairments were most frequent in perceived Cognitive Functioning, Emotional Functioning, and Fatigue, as reported in in 23 (43.4%), 22 (41.5%), and 21 (39.6%) of 53 individuals respectively. Suprathreshold Anxiety Scores were reported in 20/75 (26.7%) individuals; only 1/75 (1.3%) reported a clinically significant Depression Score.

### Factors Affecting Performance in Neurocognitive Testing Prior to Treatment

All significant predictors in univariate and multivariable models are summarized in Table 4.

**Table 4.** Predictors of Performance on Objective Neurocognitive Tests Prior to Treatment

Variable	Figure immediate recall	List recall	Figure delayed recall	List learning	Semantic l fluency	Stroop switching	Stroop interference	Digit span	Similarities	Matrix reasoning	Boston naming test	Phonemic l fluency	Information processing
Estimated Premorbid IQ	••	•••	•••	•••	•••	•••	•••	•••	•••	•••		•••	
Tumor volume	*	**		***	***				**				
Astrocytoma/GBM	**	**						***					
Self-reported depression score	***		***	*									
Self-reported cognitive functioning	••		••	•••									
Medical history burden	*						***			**			
Use of dexamethasone					***	***							
Tumor in left temporal lobe		***									**		
Age	**	*											
History of GTCS											•••		
WHO Grade		***											
Self-reported role functioning	•		•	•									
Self-reported emotional functioning	•		•										
Self-reported fatigue score	*					*							
Presence of IDH mutation		•											
Self-reported social functioning	•												
Sex													
History of any seizure													
Use of levetiracetam													
Tumor in language-dominant hemisphere													
Tumor in left frontal lobe													
Tumor in right frontal lobe													
Tumor in right temporal lobe													
Self-reported anxiety score													
Self-reported physical functioning													
Insular involvement %													

•/* Predictor of better/worse performance in univariate model only.

••/** Predictor of better/worse performance in multivariable model (not significant).

•••/*** Predictor of better/worse performance in multivariable model (significant).

#### Language.—

For BNT performance (*n* = 60), history of generalized tonic-clonic seizures (GTCS) and a left temporal lobe tumor explained 13.3% of the variance in test scores. History of GTCS predicted better performance (β = 0.97, 95% CI [0.21, 1.75], *P* = .014), and was the only significant predictor. The presence of a left temporal lobe tumor showed a nonsignificant trend toward worse performance (β = −0.73, 95% CI [−1.49, 0.04], *P* = .064). In the Similarities test, 32.3% of the variance in performance was explained by tumor volume and estimated premorbid IQ. Only the latter was a significant predictor, with a higher IQ being associated with better test performance (β = 0.04, 95% CI [0.03, 0.06], *P* < .001). On the Semantic Verbal Fluency test (*n* = 86), treatment with dexamethasone, tumor volume, and estimates of premorbid IQ collectively explained 17.8% of the variance in preoperative test scores. Dexamethasone use (β = −0.93, 95% CI [−1.59, −0.27], *P* = .007) and greater tumor volume (β = −0.006, 95% CI [−0.013, −0.000006], *P* = 0.048) both predicted worse performances, whereas a higher estimated premorbid IQ was associated with better test scores (β = 0.03, 95% CI [0.007, 0.054], *P* = .011).

#### Memory.—

On word List Recall (*n* = 80), 31.7% of the variance was explained by tumor-related factors and estimates of premorbid IQ. Left temporal lobe tumors and higher WHO grade were both significant predictors of worse test scores (β = −1.02, 95% CI [−1.58, −0.48], *P* < .001; and β = −0.39, 95% CI [−0.78, −0.01], *P* = 0.047), while greater premorbid IQ was a significant predictor of better performance (β = 0.03, 95% CI [0.01, 0.06], *P* = .004). Tumor volume and a diagnosis of astrocytoma/GBM were not significant predictors in the model, but contributed to the variance. In Figure Immediate Recall (*n* = 46), 39.5% of variance was explained by age, astrocytoma/GBM diagnosis, estimated premorbid IQ, self-reported Depression, and perceived Cognitive Functioning scores. Depression Score was the only significant predictor of performance, with greater symptom burden being associated with worse test scores (β = −0.15, 95% CI [−0.26, −0.04], *P* = .012). Furthermore, 30.3% of the variance in Figure Delayed Recall (*n* = 49) scores was explained by estimated premorbid IQ, self-reported Depression and Cognitive Functioning scores. Depression Score was also significant predictor of performance in the Figure Delayed Recall test, with greater Depression Scores predicting worse performance (β = −0.11, 95% CI [−0.20, −0.02], *P* = .022). In contrast, higher premorbid IQ was significantly associated with better performance (β = 0.03, 95% CI [0.01, 0.05], *P* = .008). Self-reported Cognitive Functioning was not a significant predictor.

#### Verbal Learning.—

Tumor volume, estimated premorbid IQ, and self-reported Cognitive Functioning were all significant predictors of List Learning test scores, explaining 58.3% of the variance (*n* = 50). Greater tumor volume was associated with a decrease in performance (β = −0.02, 95% CI [−0.03, −0.01], *P* < .001), whereas greater premorbid IQ and perceived Cognitive Functioning both predicted better test scores (β = 0.04, 95% CI [0.02, 0.07], *P* = .001; and β = 0.02, 95% CI [0.01, 0.04], *P* < .001, respectively).

#### Executive Functioning and Information Processing.—

Variance in Phonemic Verbal Fluency performance was only explained by the estimated premorbid IQ (*n* = 85, 16.0% of variance explained), with higher estimates predicting better performance (β = 0.043, 95% CI [0.02, 0.06], *P* < .001). On the Stroop Interference task (*n* = 86), medical history burden and estimated premorbid IQ explained 12.3% of the variance in test scores. Greater medical history burden was a significant predictor of worse performance (β = −0.28, 95% CI [−0.54, −0.03], *P* = .031), whereas higher estimated premorbid IQ was associated with greater test scores (β = 0.026, 95% CI [0.008, 0.044], *P* = .005). On Stroop Switching (*n* = 54), dexamethasone use and estimated premorbid IQ explained 17.5% of the variance. Dexamethasone was again a significant predictor of worse test scores (β = −0.77, 95% CI [−1.50, −0.03], *P* = .041), while higher estimates of premorbid IQ predicted better performance (β = 0.039, 95% CI [0.013, 0.065], *P* = .004). On the Digit Span test (*n* = 79), 34.5% of the variance in scores was explained by histopathological diagnosis and estimated premorbid IQ. Diagnosis of an astrocytoma/GBM was a significant predictor of worse performance (β = −0.51, 95% CI [−0.89, −0.13], *P* = .010), while higher estimated premorbid IQ was a significant predictor of better performance (β = 0.05, 95% CI [0.03, 0.06], *P* < .001).

#### Information Processing and Nonverbal Reasoning.—

No significant predictors were identified for performance on Information Processing. For the Matrix Reasoning test, 25.6% of the variance in performance was explained by medical history burden and estimated premorbid IQ. While estimated premorbid IQ was the only significant predictor, (β = 0.04, 95% CI [0.03, 0.06], *P* < .001), medical history burden was close to the threshold for significance (β = −0.23, 95% CI [−0.468, 0.004], *P* = .054).

### Agreement Between Subjective and Objective Reports of Cognitive Impairment

No significant associations were identified between preoperative objective and subjective reports of impairment across any of the 13 cognitive tests (χ2 tests or Fischer’s Exact Test *P* > .05). For illustrative purposes, this dissociation is described for verbal memory. Here, a subset of patients with significant objective impairments on List Learning appeared to be unaware of their deficits, while another subset of individuals without objective impairments self-reported themselves to be impaired (Supplementary Figure 2). Self-reported cognitive functioning was significantly correlated with Anxiety Score (*r* = −0.56, *P* < .001, *n* = 52); Depression Score (*r* = −0.60, *P* < .001, *n* = 52); and Fatigue Score (*r* = −0.58, *P* < .001, *n* = 53), see Supplementary Section 4.

## Discussion

Glioma patients are susceptible to impairments in cognition and HRQoL prior to anti-tumor treatment. In our 96 treatment-naïve brain tumour patients, up to 61.5% demonstrated cognitive impairments, while 43.4% reported subjective symptoms. Clinical-, tumor-, and patient-related factors each influenced specific cognitive functions to different degrees. A disparity emerged between subjective and objective cognitive impairment status, with objectively impaired individuals perceiving themselves to be unimpaired, and *vice versa*. In line with previous observations,^7^ patients with higher mood and fatigue scores were more likely to self-report poorer cognitive functioning. Given that self-reported scales and objective evaluations appear to capture different, complementary information about the patient’s overall performance, our results re-iterate the importance of considering both aspects to ascertain the impact of pathology and associated treatments on patient outcomes.

### Incidence of Impairments

Previous studies report a high incidence of neurocognitive impairment and subjective performance complaints in glioma patients.^10–12,22,29–31^ Our observed incidence of preoperative impairments in at least one cognitive domain (61.5%) is consistent with a systematic review that reported a median incidence of 62.6% (IQR: 31.0–79.0%).^12^ Learning, verbal memory, and language were the most susceptible domains in our cohort, followed by visual memory, executive function, and speed of information processing. A more recent study (*n* = 168)^11^ reported significant impairments in executive function and attention (42.8%), memory (34.3%), psychomotor speed (29.3%), visuospatial functioning (28.6%), and language (21.9%). While deficits in executive function were less common in our cohort, one explanation may be the less frequent frontal lobe involvement in our sample relative to theirs.^11^ In our cohort, we also observed that a substantial proportion of patients exhibited impairments in 2 or more objective tests (39.6%), even when a more stringent threshold of 2 *z*-scores ≤ -2 was considered (29.2%), reinforcing that disrupted cognitive functioning is common among treatment-naïve brain tumor patients. HRQoL complaints, especially in Cognitive and Emotional Functioning (both with an incidence > 40%), Fatigue (39.6%), and Anxiety (26.7%) were also frequent. Conversely, clinically significant levels of depression were rare, as previously reported.^10^

### Factors Contributing to Cognitive Impairment

While the link between tumor burden and pretreatment impairment has previously been described,^11,12^ large heterogeneity remains in which patients experience deficits.^1^ Compared to tumor burden, few studies have probed the contribution of medical history, premorbid ability, psychological well-being, and other factors captured via self-report scales on cognitive performance. In our cohort, a combination of clinical-, tumor- and patient-related factors accounted for up to 58.3% of variance in cognitive test performance. Tumor volume, diagnosis of Astrocytoma/GBM, self-reported Depression Score, medical history burden, and dexamethasone use were the top predictors of *worse* performance on objective neurocognitive tests. As expected, higher estimated premorbid IQ was a significant predictor of *better* performance in 10/13 tests, congruent with previous reports of IQ outperforming clinical variables when predicting language performance.^32^ Premorbid IQ did not explain variance in the BNT or Speed of Information Processing, suggesting that these tests may be sensitive to selective deficits that rely more heavily on task-specific abilities, as opposed to general intelligence. An important question that warrants further investigation is why some individuals appear more resilient to neuropathology than others. While it has been suggested that premorbid IQ may to some extent act as a proxy for the highly complex variable of “cognitive reserve,”^6,32^ there are likely many factors involved in driving such resilience, including lifestyle factors and occupational complexity.^33^ In addition to understanding detrimental factors, identifying factors that positively affect cognitive performance is also crucial to help better contextualize neuropsychological test scores.

Tumor-related factors explained substantial variance in pretreatment neurocognitive test performance, as expected. While tumor grade has previously been reported to affect objective impairments,^1,11–13^ grade was not a frequent predictor of performance in our cohort. Although lower tumor grade was not observed to positively affect performance, we cannot exclude the possibility that their slow-growing nature enabled functional reorganization.^1^ Because of the imbalanced grouping in our cohort (approximately 3:2:1 ratios of tumor Grades 2, 3, and 4), the number of patients with high-grade tumors was likely too low in some regression models for significant effects to present. An alternative explanation is the recent shift in emphasis on molecular diagnosis, wherein *isocitrate dehydrogenase* (IDH)-wildtype Grade II Astrocytomas are now instead recognized as glioblastoma. Tumor grade alone may, therefore, be less informative than tumor cell lineage, *IDH*-status or other emerging molecular markers,^34^ supported by our finding that Astrocytoma/GBM diagnosis predicted verbal and nonverbal memory performance.

Among clinical factors, a greater medical history burden, dexamethasone use, and a history of GTCS were significantly associated with worse performance on several tests. Clinical comorbidities may affect cognitive function^6^ due to independent pathophysiological processes or as a consequence of associated medications. Indeed, corticosteroid use has been associated with worse performance on memory tests,^35^ though evidence is conflicting.^22^ Dexamethasone use was not associated with memory performance in our cohort, but instead reduced performance on language and executive function tests. Such differences may partly reflect cohort dissimilarities. However, dosage and durations of dexamethasone very likely varied between initial diagnosis, neuropsychological assessment and surgery. Consequently, we cannot determine if cognitive impairment resulted from chronic versus acute dexamethasone use and/or edema prompting corticosteroid treatment.

The unexpected finding that a history of generalized seizures predicted *better* BNT performance may—speculatively—reflect the presentation of seizures potentially resulting in earlier diagnosis (ie, prior to detectable language decline). Despite previous reports of anti-epileptic drugs affecting cognitive performance in glioma patients,^6^ seizure history or levetiracetam use were not significant predictors of test performance. However, there is conflicting evidence in the literature,^36^ indicating possible participant-specific side-effect profiles or polytherapy effects.^37^ These, coupled with a reduced sample size (*n* = 58/96, 60.4%) may have prevented trends from presenting in our data.

Language-dominant hemisphere involvement did not emerge as a significant predictor of language function, likely because the majority of our neuropsychological assessments are conducted to inform surgical risks in the language-dominant hemisphere. Meaningful trends did, however, appear on a lobe level, with left temporal lobe tumors predicting worse List Recall and BNT scores. This is consistent with previous studies identifying significantly worse performance in expressive language and memory in patients with left versus right temporal lobe tumors.^22^

Several self-reported item scores were associated with cognitive performance in univariate models. However, only Depression score and perceived Cognitive Functioning remained significant in multivariable models. The influence of Depression scores reinforces the interplay between normal variations in mood and psychological state, and both subjective^38^ and objective cognitive performance. Interestingly, although high levels of anxiety were reported, these did not predict performance on any neurocognitive tests. Any detrimental influence of a documented chronic anxiety disorder on test performance^39^ may differ from the acute anxiety associated with a brain-tumor diagnosis. Future studies would benefit from evaluating trait versus state symptom burdens separately.

Supplementing mood and anxiety scales, Boele et al.,^8^ previously described correlations between self-reported HRQoL items and objective cognitive performance, with physical- and mental health scores both correlating with several cognitive domains. While Physical Functioning did not predict performance in our cohort, our and Boele’s^8^ findings highlight the utility of self-report scales in capturing critical aspects of the patients’ overall functioning. In our cohort, there was a lack of agreement between objective and subjective measures of impairment status across all tests, similar to previous trends.^5,7^ For example, in the List Learning test (illustrated in Supplementary Figure 2), 7/52 (13.5%) patients with objective impairments self-reported themselves to be unimpaired. This discrepancy may be due to limited self-awareness,^7^ or because patients may not have perceived a large deviation from their premorbid baseline (eg, due to slow, gradual declines). Conversely, 13/52 (25.0%) of patients with subjective complaints were not considered objectively “impaired.” These findings reflect limitations in the ecological validity of some neuropsychological tests, which potentially have low sensitivity to certain aspects of functioning that substantially impact quality of life. In addition, some patients with high premorbid IQ may experience cognitive deterioration that is not objectively captured if their scores remain within the average range. Given the clinical and scientific importance of monitoring and understanding treatment effects on cognition, these findings highlight that an absence of self-reported complaints should not rule out referral for objective neuropsychological testing, and *vice versa*.

Importantly, while our preselected factors meaningfully explained variability in cognitive test performance, between 41.7% and 87.7% of variance in performance on individual tests remained unaccounted for. Further studies are warranted to explore additional factors such as emerging intratumoral molecular markers,^34^ neuroimaging metrics of white matter integrity and/or functional connectivity,^1^ more granular patient-specific factors (eg, performance status, comorbidities, medications, environmental factors), and multi-dimensional fatigue scales.

### Study Limitations

While participants were not selected by tumor location, hemisphere, or surgery type,^12^ selection bias may have occurred through the preponderance of language-dominant gliomas. Moreover, the high prevalence of language-dominant hemisphere tumors in our cohort reflects the typical prioritization of these patients for neuropsychological assessment. Consequently, the number of patients with tumors in other locations may have been too low for expected location-based effects to emerge. As the global initiative to develop shorter, standardized batteries for glioma patients continues, we hope that this problem will be mitigated in future studies, to help better address the under-representation of other tumor locations across the literature. Given that clinical neuropsychological assessments are often performed in a hypothesis-driven manner, responding to the clinicians’, patients’, and/or carers’ reports, our results may also overestimate impairments relative to the general glioma population. In addition, while we aimed to characterize the influence of medical history burden on cognition, our retrospective study was limited to information available in the neurosurgical notes. Consequently, we could not account for how well-controlled patients’ comorbid conditions were, nor how long the patients had experienced each condition. These factors may affect preoperative cognitive performance and warrant further research. Furthermore, while age-specific normative data were used to generate *z*-scores, level of education was not typically adjusted for in most tests (except for BNT and Phonemic Verbal Fluency) as part of our routine clinical practice. It is therefore possible that education level, as well as other factors such as socioeconomic status, environment, and occupational status/complexity may have influenced performance on objective tests in our cohort. Finally, given the large heterogeneity of glioma populations, interpretations of the conclusions from this study of a smaller cohort warrants caution, and strongly motivates the need for large-scale studies to be performed.

## Conclusion

In conclusion, glioma patients exhibit objective neurocognitive and self-reported impairments across several domains prior to anti-tumor treatment. Along with tumor burden, our study highlights the influence of the patient’s premorbid ability, medical history, and mental health on neurocognitive test performance, reinforcing the importance of considering wider factors when interpreting pre- and post-treatment performance. Additional factors, such as extent of white matter infiltration and molecular markers, likely contribute to the remaining unexplained variance in cognitive performance and outcomes among glioma patients. Crucially, the disparity between subjective and objective metrics of cognitive impairment reinforces the importance of combining neuropsychological and HRQoL assessments to fully evaluate the impact of gliomas and their treatments on patient outcomes.

## Supplementary material

Supplementary material is available online at *Neuro-Oncology Practice* (https://academic.oup.com/nop/).

npae106_suppl_Supplementary_Material_S4

npae106_suppl_Supplementary_Material_S3

npae106_suppl_Supplementary_Material_S1

npae106_suppl_Supplementary_Table_S1

npae106_suppl_Supplementary_Figure_S1

npae106_suppl_Supplementary_Figure_S2

npae106_suppl_Supplementary_Material_S2

npae106_suppl_Supplementary_Table_S2

## Data Availability

All composite scores (incidence, mean and SD scores) on which statistics were based are presented in the manuscript and supporting information. Individual de-identified *z*-scores are available from the corresponding author upon reasonable request.
