# Supplementary material for: Factors contributing to variability in neurocognitive performance before glioma neurosurgery
Source: Neurooncol Pract. 2024 Oct 20;12(2):301–12. doi: 10.1093/nop/npae106 (PMC11913645; doi:10.1093/nop/npae106)
Supplement: npae106_suppl_Supplementary_Material_S4 [file npae106_suppl_supplementary_material_s4.docx]

**SUPPLEMENTARY MATERIALS**

**Incidence of Post-operative Impairment & Changes**

Forty-five patients with radiologically stable disease underwent post-surgical neuropsychological assessment, on average 331.2 (± 190.9) days after surgery. Of these, 44 patients had both pre- and post-operative data available.

**Method**

Incidence of post-operative impairments are reported for all cognitive tests (Manuscript Table 1). In one patient, the California Verbal Learning test was performed pre- and post-operatively for the list learning task, instead of using the BMIPB list.

To evaluate significant post-operative change, reliable change indices (RCIs) ^1^ were calculated for seven neurocognitive assessments, with correction for regression to the mean, as follows: *Corrected Score 1 = (Score 1 – Mean 1) * Reliability + Mean 1*. As per standard practice, a 90% confidence interval ^1^ was used to detect meaningful deterioration ( RCI ≤ -1.645) or improvement (≥ +1.645 . To calculate RCIs, we consulted the raw or standard scores, which were only available for 7 out of the 13 cognitive tests. Unfortunately, for the other 6 tests the required data were not systematically transcribed in the result summaries. Demographics about this cohort can be viewed below in Table 1 (below).

**Incidence of Significant Post-Operative Impairments and Changes**

Objective and subjective impairments were common post-operatively (Figure 1). The incidence and directionality of significant changes are shown in Figure 2. Learning, language, and memory were most commonly affected, followed by information processing and executive functioning.

| **Table 1.** **Demographics of the Post-operative Cohorts** | |
| --- | --- |
|  | **Post-operative Cohort** |
| **N** | 45 |
| **Age** (years), mean ± SD | 38.7 ± 12.7 |
| **Sex** (male:female), n (%) | 13:22 (51.1%:48.9%) |
| **Tumour Type**, n (%) |  |
| *IDH^mut^* Astrocytoma |  |
| WHO Grade 2 | 12 (26.7%) |
| WHO Grade 3 | 10 (22.2%) |
| WHO Grade 4 | 0 (0%) |
| *IDH^mut^* Oligodendroglioma |  |
| WHO Grade 3 | 12 (26.7%) |
| WHO Grade 4 | 4 (8.9%) |
| *IDH^WT^* Glioblastoma | 5 (11.1%) |
| Not Otherwise Specified | 2 (4.4%) |
| **Tumour Location,** n (%) |  |
| Left Hemisphere *Frontal* | 13 (28.9%) |
| *Parietal* | 1 (2.2%) |
| *Temporal* | 16 (35.6%) |
| *Insular (isolated)* | 1 (2.2%) |
| Right Hemisphere *Frontal* | 10 (22.2%) |
| *Parietal* | 1 (2.2%) |
| *Temporal* | 1 (2.2%) |
| *Insular (isolated)* | 1 (2.2%) |
| Both Hemispheres | 1 (2.2%) |
| **Insular Involvement ^a^,** n (%) | 9 (20.0%) |
| **Previous Biopsy,**  Yes : No n (%) | 1 : 44 (2.2% : 97.8%) |
| **Pre- to Post-operative Neuropsychology ^b^**, days (mean ± SD) | 476 ± 338 |
| **Post-operative Radiotherapy,** yes/no/unknown (%)  **Post-operative Chemotherapy,** yes/no/unknown (%) | 51.1% : 33.3% : 15.6%  40.0% : 37.8% : 22.2% |
| \| Tumour in Language-dominant hemisphere? \|  \| \| --- \| --- \| \| *Yes* \| 33 (73.3%) \| \| *No* \| 12 (26.7%) \| \| *Bilateral / likely typical / unconfirmed* \| - \| \| Use of Dexamethasone, n (%) \|  \| \| *Yes* \| 7 (15.6%) \| \| *No* \| 38 (84.4%) \| \| Use of anti-epileptic drugs, n (%) \|  \| \| *Yes* \| 33 (73.3%) \| \| *No* \| 12 (26.7%) \| \| Use of Levetiracetam, n (%) \|  \| \| *Yes* \| 25 (55.6%) \| \| *No* \| 20 (44.4%) \| \| Number of medicated comorbidities, n (%) \|  \| \| *0* \| 29 (64.4%) \| \| *1* \| 9 (20.0%) \| \| *≥2* \| 7 (15.6%) \| \| *Time between surgery and follow-up neuropsychology, mean ± SD (days)* \| 331.2 ± 190.9 \| \| *Estimate of Premorbid IQ, mean ± SD* \| 103.0 ± 11.0 \| | |

**
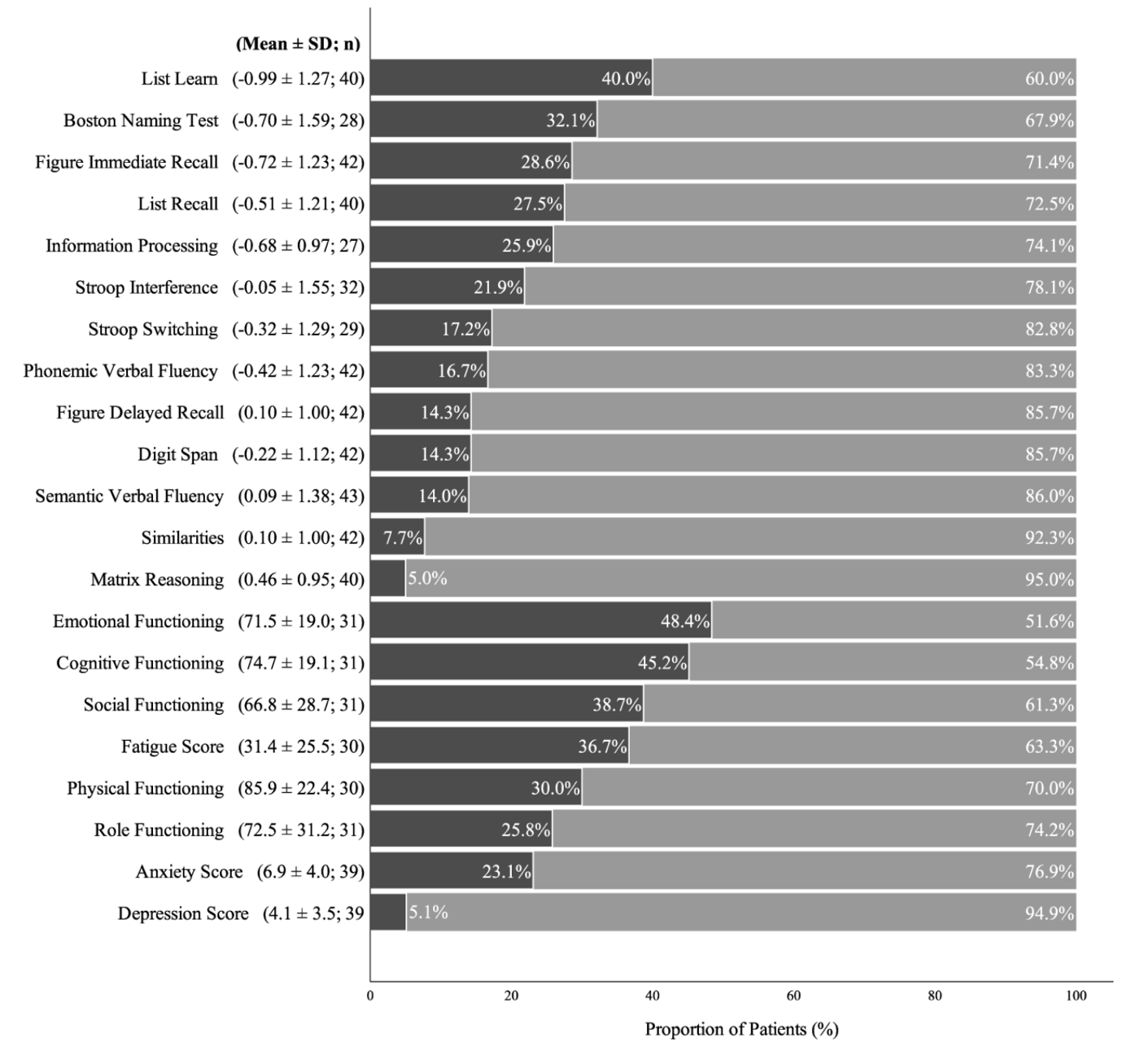
**

**Figure 1. Incidence of Post-operative Impairments on Objective Cognitive Tests and Self-reported Scales**. The percentage of patients (out of total available, ‘n’) that are impaired (darker grey) versus not impaired (lighter grey) are shown, with corresponding mean and standard deviation (SD) of z-scores. Overall, post-operative impairments were observed across all tests and scales. Memory and language were the most frequently impaired cognitive domains, whereas perceived Emotional-, Cognitive-, and Social Functioning were the most frequently affected self-reported items.

Clinically significant deterioration in any domain occurred in 25/43 (58.1%) patients, 8/43 (18.6%) improved, whilst 10/43 (23.3%) did not change significantly. On a test-level, significant deterioration and improvement, respectively, were as follows: List Learning (n=12/39, 30.8% deteriorated | none improved); Stroop Interference (n=6/29, 20.7% | none); Semantic Verbal Fluency (n=6/43, 14.0% | n=1/43, 2.3%); BNT (n=4/29, 13.8% | n=1/29, 3.4%); Digit Span (n=5/43, 11.6% | n=1/43, 2.3%); Stroop Switching (n=3/26, 11.5% | none); Phonemic Verbal Fluency (n=4/42, 9.5% | 4/42, 9.5%); Information processing (n=2/24, 8.3% | 1/24, 4.2%). Only 11/43 (25.6%) patients deteriorated in ≥ 2 tests. No patients improved in >1 task. RCIs could not be calculated for the self-reported metrics. However, where both pre- and post-operative data were available, post-operative changes in impairment status were observed (Table 2 below).


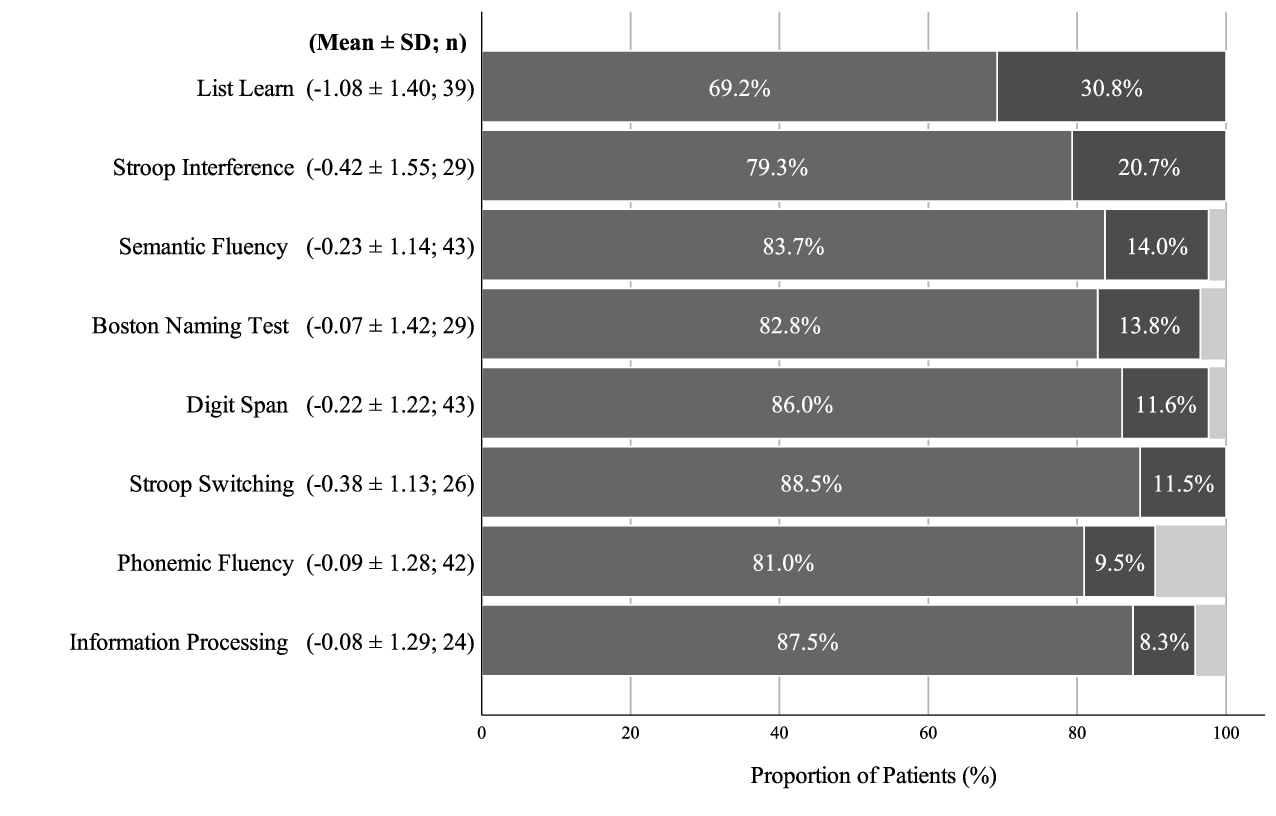


**Figure 2. Incidence of Significant Changes in Post-operative Performance on Objective Neurocognitive Tests in Brain Tumour Patients.** Clinically meaningful changes in cognitive scores were determined using reliable change indices (RCIs), with an RCI of ≤ -1.645 indicating deterioration and an RCI ≥ +1.645 indicating improvement. Following surgery, 25/43 (58.1%) patients deteriorated in at least one domain, 8/43 (18.6%) improved, whilst 10/43 (23.3%) did not significantly change. Deterioration was most frequent in memory, executive function, and language tests. The rates of improvements on individual tests were: semantic fluency (2.3%), Boston Naming Test (3.4%), Digit Span (2.3%), Phonemic Verbal Fluency (9.5%), and Information Processing (4.2%). No improvements were identified in the other tests.

| Table 2. Changes in Impairment Status Following Surgery. | | | |
| --- | --- | --- | --- |
|  | **Post-operative change in Impairment Status** | | |
| Item | **New Impairment**  **n (%)** | **No Longer Impaired**  **n (%)** | **No change in status, n (%)** |
| Anxiety Score | 6 (16.2%) | 4 (10.8%) | 27 (73.0%) |
| Depression Score | 2 (5.4%) | - | 35 (94.6%) |
| Physical Functioning | 4 (16.7%) | - | 20 (83.3%) |
| Role Functioning | 2 (8.0%) | 4 (16.0%) | 19 (76.0%) |
| Emotional Functioning | 8 (32.0%) | 4 (16.0%) | 13 (52.0%) |
| Cognitive Functioning | 4 (16.0%) | 6 (24.0%) | 15 (60.0%) |
| Social Functioning | 6 (24.0%) | - | 19 (76.0%) |
| Fatigue | 4 (16.0%) | 2 (8.0%) | 19 (76.0%) |

A preliminary analysis found there to be a greater incidence of declines in patients who had chemoradiation after surgery in our cohort (X^2^=5.7; df=1, p=0.017); 14/18 (77.8%) patients who underwent chemoradiation declined in at least one test post-operatively, whilst 6/16 (37.5%) of those who did not receive chemoradiation declined (1 of these received RT only, and also declined). Information about post-operative treatments was not available for the remaining 10 patients.

**Discussion**

Many patients deteriorated post-operatively in at least one test (58.1%). Memory, executive function, and language tests were most commonly affected. However, declines in ≥ 2 tests were less frequent (25.6%). Nearly half of the patients underwent post-operative radiotherapy and/or chemotherapy prior to the follow-up neuropsychology appointment. In line with what we observed, these adjuvant therapies are known to affect cognitive function in glioma patients ^2^. Consequently, the extent to which longitudinal changes resulted from surgery as opposed to adjuvant treatment could not be disentangled. A recent meta-analysis ^3^ highlighted that, overall, glioma surgery can have beneficial effects on cognition. Due to our limited longitudinal sample, we could not explore the contribution of different variables to post-surgical cognition. Hypothetically, alleviation of tumour-related tissue compression / swelling would be expected to improve post-operative function; while progressive tumour infiltration, surgical complications and destruction of functionally relevant tissue likely also contribute to post-operative decline.

**Limitations**

In the post-operative cohort, follow-up timings were variable and many were lost to follow-up. As OUH is a specialist neuro-oncology surgery centre with a large catchment area, many patients undergo adjuvant therapy closer to home. Numerous patients could not be reached or declined invitations to return. It is possible that patients without subjectively perceived deficits choose not to return, or that neurocognitive follow-up is de-prioritised in the context of their wider diagnosis, a factor which may shift over time as treatments become better at prolonging survival.

**References**

1. Iverson GL, Sawyer DC, McCracken LM, Kozora E. Assessing depression in systemic lupus erythematosus: determining reliable change. *Lupus*. 2001;10(4):266-271. doi:10.1191/096120301680416959

2. Kirkman MA, Hunn BHM, Thomas MSC, Tolmie AK. Influences on cognitive outcomes in adult patients with gliomas: A systematic review. *Front Oncol*. 2022;12. doi:10.3389/fonc.2022.943600

3. Ng JCH, See AAQ, Ang TY, Tan LYR, Ang BT, King NKK. Effects of surgery on neurocognitive function in patients with glioma: a meta-analysis of immediate post-operative and long-term follow-up neurocognitive outcomes. *J Neurooncol*. 2019;141(1):167-182. doi:10.1007/s11060-018-03023-9
