# Supplementary material for: Factors contributing to variability in neurocognitive performance before glioma neurosurgery
Source: Neurooncol Pract. 2024 Oct 20;12(2):301–12. doi: 10.1093/nop/npae106 (PMC11913645; doi:10.1093/nop/npae106)
Supplement: npae106_suppl_Supplementary_Material_S3 [file npae106_suppl_supplementary_material_s3.docx]

**SUPPLEMENTARY MATERIALS**

**Thresholds for Clinically Significant Impairment**

*EORTC QLQ-C30:*
<83 for Physical Functioning, <58 for Role functioning, <58 for Social Functioning, <71 for Emotional functioning, <75 for Cognitive Functioning, and >39 for Fatigue ^1^.

*HADS:*
Scores ≥ 11 in the anxiety or depression subscales indicate a likely presence of clinically significant mood disorder ^2^.

1. Giesinger JM, Loth FLC, Aaronson NK, et al. Thresholds for clinical importance were established to improve interpretation of the EORTC QLQ-C30 in clinical practice and research. *J Clin Epidemiol*. 2020;118:1-8. doi:10.1016/j.jclinepi.2019.10.003

2. Zigmond AS, Snaith RP. The Hospital Anxiety and Depression Scale. *Acta Psychiatr Scand*. 1983;67(6):361-370. doi:10.1111/j.1600-0447.1983.tb09716.x
