# Supplementary material for: Factors contributing to variability in neurocognitive performance before glioma neurosurgery
Source: Neurooncol Pract. 2024 Oct 20;12(2):301–12. doi: 10.1093/nop/npae106 (PMC11913645; doi:10.1093/nop/npae106)
Supplement: npae106_suppl_Supplementary_Material_S1 [file npae106_suppl_supplementary_material_s1.docx]

**SUPPLEMENTARY MATERIALS**

**Illustrative Example of Multivariable Regression with Backward Selection**

Depression and anxiety may both be correlated with memory performance. In backward selection, Depression Scores would first be excluded from the multivariable model, then Anxiety Scores separately. If the goodness of fit of the model for memory performance is better when Depression Scores are excluded rather than Anxiety Scores, the implication is that Anxiety Scores are more relevant for understanding memory.
