# Supplementary material for: Factors contributing to variability in neurocognitive performance before glioma neurosurgery
Source: Neurooncol Pract. 2024 Oct 20;12(2):301–12. doi: 10.1093/nop/npae106 (PMC11913645; doi:10.1093/nop/npae106)
Supplement: npae106_suppl_Supplementary_Table_S1 [file npae106_suppl_supplementary_table_s1.docx]

**SUPPLEMENTARY MATERIALS**

| Supplementary Table 1. Additional Demographic Data for Pre-Operative Cohorts | | | | |
| --- | --- | --- | --- | --- |
|  | | **Pre-operative Cohort**  **N = 96** | | |
| Tumour in Language-dominant hemisphere? | |  | | |
| *Yes* | | 62 (64.6%) | | |
| *No* | | 31 (32.3%) | | |
| *Bilateral / likely typical / unconfirmed* | | 3 (3.1%) | | |
| Use of Dexamethasone, n (%) | |  | | |
| *Yes* | | 18 (18.8%) | | |
| *No* | | 78 (81.3%) | | |
| Use of anti-epileptic drugs, n (%) | |  | | |
| *Yes* | | 73 (76.0%) | | |
| *No* | | 23 (24.0%) | | |
| Use of Levetiracetam, n (%) | |  | | |
| *Yes* | | 58 (60.4%) | | |
| *No* | | 38 (39.6%) | | |
| Number of medicated comorbidities, n (%) | |  | | |
| *0* | | 54 (56.3%) | | |
| *1* | | 23 (24.0%) | | |
| *≥2* | | 19 (19.8) | | |
| Estimate of Premorbid IQ, mean ± SD | | 101.8 ± 11.4 | | |
| Occupation |  | |  |  |
| *Academic (student or professional)* |  | | 3 (3.1%) |  |
| *Domestic (housewife/carer)* |  | | 2 (2.1%) |  |
| *Health sector* |  | | 7 (7.3%) |  |
| *Office-based Work* |  | | 21 (21.9%) |  |
| *Other professions* |  | | 8 (8.3%) |  |
| *Practical Work (including physical/specific expertise)* |  | | 23 (24.0%) |  |
| *Retail/Business* |  | | *15 (15.6%)* |  |
| *Education* |  | | *8 (8.3%)* |  |
| *Retired or Unemployed* |  | | *3 (3.1%)* |  |
| *Not Available* |  | | 6 (6.3%) |  |
