# Supplementary material for: Factors contributing to variability in neurocognitive performance before glioma neurosurgery
Source: Neurooncol Pract. 2024 Oct 20;12(2):301–12. doi: 10.1093/nop/npae106 (PMC11913645; doi:10.1093/nop/npae106)
Supplement: npae106_suppl_Supplementary_Figure_S1 [file npae106_suppl_supplementary_figure_s1.docx]

**SUPPLEMENTARY MATERIALS**

**Incidence Of Impairment Per Objective Cognitive Test, Organised By Primary Lobe Affected (z ≤ -1.5)**

**Key:**

FL = Frontal lobe – left

FR = Frontal lobe – right

PL = Parietal lobe – left

PR = Parietal lobe – right

TL = Temporal lobe – left

TR = Temporal lobe – right

IL = Insular lobe – left

IR = Insular lobe – right

**Note**: As no patients with a right insular tumour had data for the Stroop Switching task, this bar was omitted and the corresponding plot below subsequently has its own, modified scale (italic).

0

5

20

30

0

10

20

30

0

10

20

30

0

10

20

30

0

10

20

30

0

10

20

30

0

10

20

30

0

10

20

30

0

10

20

30

0

10

20

30

0

10

20

30

**
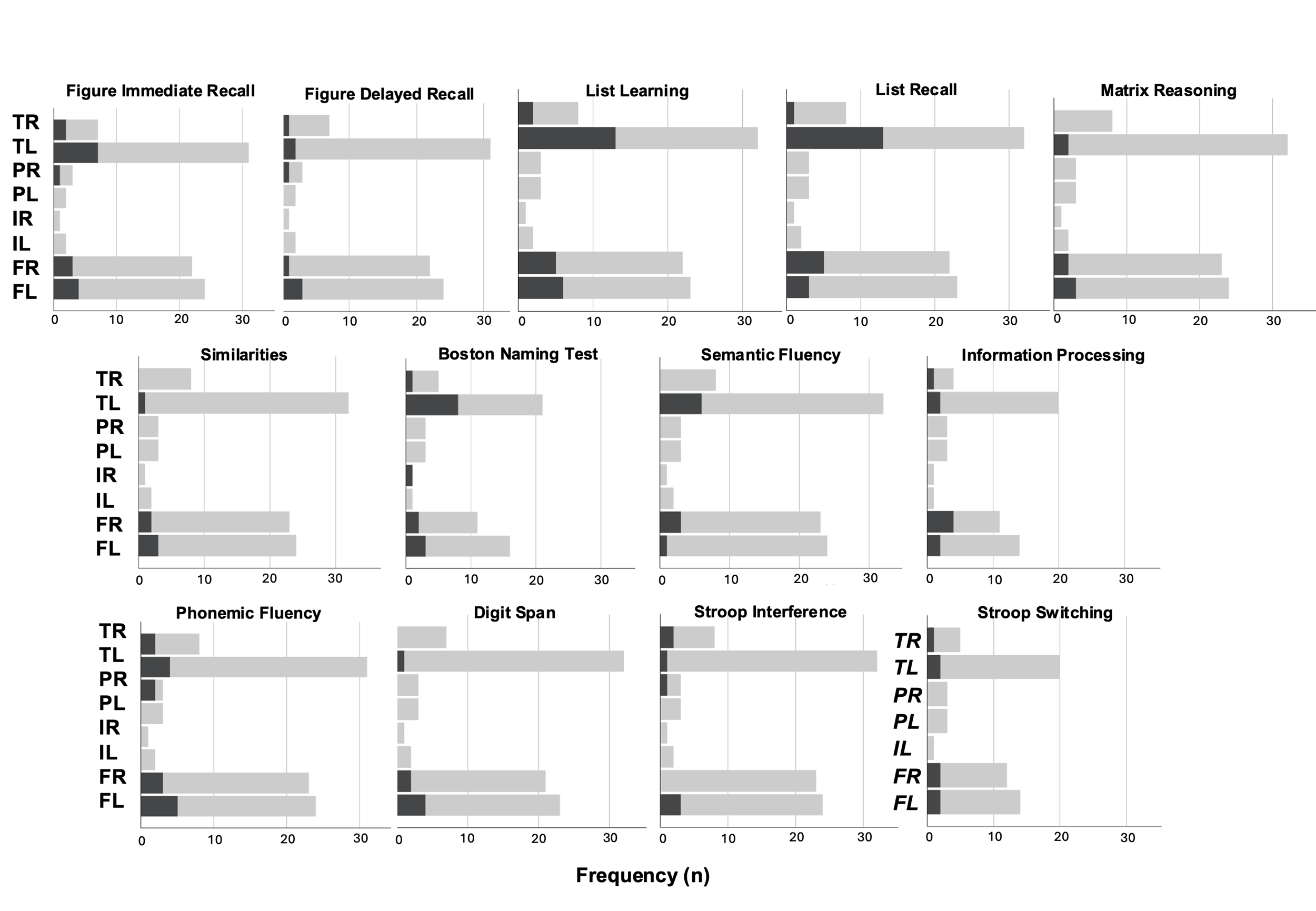
**
