# Supplementary material for: Factors contributing to variability in neurocognitive performance before glioma neurosurgery
Source: Neurooncol Pract. 2024 Oct 20;12(2):301–12. doi: 10.1093/nop/npae106 (PMC11913645; doi:10.1093/nop/npae106)
Supplement: npae106_suppl_Supplementary_Figure_S2 [file npae106_suppl_supplementary_figure_s2.docx]

**SUPPLEMENTARY MATERIALS**

**
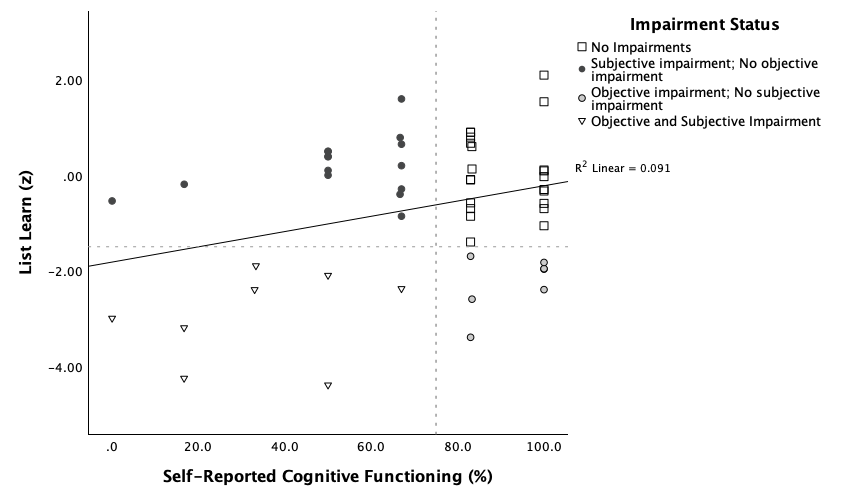
**

**Overall correlation***r = 0.301*

*Threshold for subjective impairment (<75)*

N=4

N=2

**Incongruent**

*N=7*

*Threshold for objective impairment (<-1.5)*

**Incongruent***N=13*

N=2

**Supplementary Figure 2. Correlation between performance on List Learning Task and perceived, self-reported Cognitive Functioning (n=52).** Pearson’s correlation coefficient for overall correlation = 0.301 (p=0.030). Seven individuals (13.5%; note: there are overlapping datapoints on the plot) *with* objective cognitive impairment on the list-learn task self-reported themselves to be unimpaired, whilst 13 individuals (25%) *without* objective cognitive impairment self-reported themselves to be impaired. As such, only 61.5% of patients exhibited congruence between self- and objectively reported cognitive impairment.
