# Supplementary material for: Factors contributing to variability in neurocognitive performance before glioma neurosurgery
Source: Neurooncol Pract. 2024 Oct 20;12(2):301–12. doi: 10.1093/nop/npae106 (PMC11913645; doi:10.1093/nop/npae106)
Supplement: npae106_suppl_Supplementary_Material_S2 [file npae106_suppl_supplementary_material_s2.docx]

**SUPPLEMENTARY MATERIALS**

|  | | Self-reported Cognitive functioning | Anxiety Score | Depression Score | Fatigue Score |
| --- | --- | --- | --- | --- | --- |
| Self-reported Cognitive functioning | Pearson Correlation | 1 | -.555^**^ | -.597^**^ | -.577^**^ |
|  | Sig. (2-tailed) |  | <.001 | <.001 | <.001 |
|  | N | 53 | 52 | 52 | 53 |
