# Supplementary material for: Factors contributing to variability in neurocognitive performance before glioma neurosurgery
Source: Neurooncol Pract. 2024 Oct 20;12(2):301–12. doi: 10.1093/nop/npae106 (PMC11913645; doi:10.1093/nop/npae106)
Supplement: npae106_suppl_Supplementary_Table_S2 [file npae106_suppl_supplementary_table_s2.docx]

**SUPPLEMENTARY MATERIALS**

| Supplementary Table 2. Data evaluated in linear regression models. | | |
| --- | --- | --- |
| Test | N | Predictors |
| Figure Immediate Recall | 46 | Age (range: 18-65)  Astro/GBM (17 x yes; 29 x no)  Premorbid IQ (range: 81-122)  Depression Score (range: 0-12)  Cognitive Functioning (range: 0-100) |
| Figure Delayed Recall | 49 | Premorbid IQ (range: 81-122)  Depression Score (range: 0-12)  Cognitive Functioning (range: 0-100) |
| List Learn | 50 | Tumour volume (range: 0.41 – 159 mm3)  Premorbid IQ (range: 81 – 122)  Cognitive Functioning (range: 0 -100) |
| List Recall | 90 | Left temporal lobe tumour (29 x yes; 61 x no)  Tumour volume (range: 0.41 – 172.8 mm3)  WHO Grade (43 x Grade2; 34 x Grade 3; 13 x Grade 4) Astro/GBM vs oligo (57 x yes; 33 x no) |
| Semantic Verbal Fluency | 86 | Dexamethasone (17 x yes; 69 x no)  Tumour Volume (range: 0.41 – 172.8 mm3)  Premorbid IQ (range: 70-126) |
| Phonemic Verbal Fluency | 85 | Premorbid IQ (range: 70-126) |
| Stroop Interference | 86 | Medical history burden (49 x none; 20 x 1 condition/event; 17 x 2 or more conditions/events)  Estimate of premorbid IQ (range: 70-126) |
| Stroop Switching | 54 | Dexamethasone (9 x yes; 45 x no)  Premorbid IQ (range: 81-122) |
| Digit Span | 79 | Astro/GBM (51 x yes; 28 x no)  Estimate of Premorbid IQ (range: 70 -126) |
| Boston Naming Test | 60 | History of GTCS (21 x yes; 39 x no)  Left temporal lobe tumour (21 x yes; 31 x no) |
| Matrix Reasoning | 86 | Medical history burden (49 x none; 20 x one condition/event; 17 x 2 or more conditions/events)  Premorbid IQ (70 – 126) |
| Similarities | 85 | Tumour volume (range: 0.41 – 172.8 mm3)  Premorbid IQ (range: 70-126 range) |
| Information Processing |  | None |
